# Clinical Trials of Immunogene Therapy for Spontaneous Tumors in Companion Animals

**DOI:** 10.1155/2014/718520

**Published:** 2014-11-17

**Authors:** Gerardo Claudio Glikin, Liliana María Elena Finocchiaro

**Affiliations:** Unidad de Transferencia Genética, Instituto de Oncología “Ángel H. Roffo”, Universidad de Buenos Aires, 1417 Buenos Aires, Argentina

## Abstract

Despite the important progress obtained in the treatment of some pets' malignancies, new treatments need to be developed. Being critical in cancer control and progression, the immune system's appropriate modulation may provide effective therapeutic options. In this review we summarize the outcomes of published immunogene therapy veterinary clinical trials reported by many research centers. A variety of tumors such as canine melanoma, soft tissue sarcomas, osteosarcoma and lymphoma, feline fibrosarcoma, and equine melanoma were subjected to different treatment approaches. Both viral and mainly nonviral vectors were used to deliver gene products as cytokines, xenogeneic tumor associated antigens, specific ligands, and proapoptotic regulatory factors. In some cases autologous, allogenic, or xenogeneic transgenic cytokine producing cells were assayed. In general terms, minor or no adverse collateral effects appeared during this kind of therapies and treated patients usually displayed a better course of the disease (longer survival, delayed or suppressed recurrence or metastatic spread, and improvement of the quality of life). This suggests the utility of these methodologies as standard adjuvant treatments. The encouraging outcomes obtained in companion animals support their ready application in veterinary clinical oncology and serve as preclinical proof of concept and safety assay for future human gene therapy trials.

## 1. Introduction

Gene therapy provides novel strategies to treat many cancers that lack suitable approved treatments. Cancer gene therapy clinical trials started significantly earlier in human beings [1] than in companion animals [2]. Both trials involved immunogene therapy that is based on the expression of transgenes engaged in immune responses [3].

Although the convenience of comparative oncology for translational approaches was strongly encouraged for many years [4, 5], until now (June 2014) there was a significantly higher number of human (1331) than companion animal (48) cancer gene therapy trials (http://www.wiley.com/legacy/wileychi/genmed/clinical/).

Manipulating the immune system in order to induce clinically relevant responses against cancer is a longstanding goal, and gene transfer offers a suitable approach to reach it.

As it happens in human clinical trials, cancer immunogene therapy is largely leading the major attention in companion animal trials. Most of the veterinary patients were canine [6], while some reports were derived from feline and equine patients [7]. Different kinds of tumors were targeted, being highly aggressive spontaneous canine melanoma the preferred model. Both viral [8, 9] and nonviral [10] vectors as well as therapeutic gene producing transgenic cells were used to deliver gene products such as cytokines, suicide genes, tumor antigens, and proapoptotic genes [6, 7, 11]. In the last decade, the advances in physical and chemical methods to deliver plasmid DNA into tumor tissue and normal tissue are showing promise in narrowing the gap between viral and nonviral gene delivery efficiency [12].

We focused this review only on reports of clinical data collected from client-owned patients with spontaneously arising tumors. Canine, feline, and equine trials were separately displayed in Tables 1, 2, and 3.

## 2. Canine Melanoma

Spontaneous canine melanoma is a highly aggressive tumor of the oral cavity, digit/footpad, and mucocutaneous junctions; it appears clinically similar to aggressive human melanoma. Both diseases are chemo- and radioresistant, do not respond well to treatment with conventional biological response modifiers, and share similar metastatic phenotypes and site selectivity [4, 5]. At the time of diagnosis, the disease is often metastatic and has an extremely poor prognosis because of rapid invasion of surrounding normal tissue and high likelihood of regional and distant metastasis early in the course of the disease. The high metastatic rate observed with this disease and the inefficacy of current therapies warranted the investigation into novel therapies.

The pioneering work published about 18 years ago [2] described an* ex vivo* genetically engineered xenogeneic cell gene therapy approach where the effects of repeated peritumoral local injections of human interleukin-2 (hIL-2) secreting Vero cells after surgical removal of the tumor followed by ^60^Co-radiotherapy were evaluated. This combined treatment resulted in a considerably higher median survival of 270 days compared to 75 days of the surgery plus radiation controls (*n* = 16, each group). The treatment resulted was safe.

A couple of years later, an individually targeted autologous* ex vivo* approach for spontaneous canine melanoma involving human granulocyte and macrophage colony stimulating factor (hGM-CSF) gene transfer was reported [13]. In this case, after tumor removal, a suspension of tumor cells was subjected to a gene gun shot with gold particles coated with a plasmid carrying the human GM-CSF gene. After sublethal ^137^Cs irradiation, transgenic cells were delivered as intradermal injections in the flank of the patients. As part of phase I clinical trial, the treatment of 10 canine melanoma bearing patients resulted in 3 objective responses and 1 stable disease, while 3 cases of fibrosarcoma yielded 2 objective responses. No animal exhibited any signs of local or systemic toxicity. Even though it is statistically nonsignificant because the low number of cases, survival time of melanoma responders (>300 days) largely exceeded the expected values for surgery treated patients (90–165 days).

By using intratumoral injections of bacterial superantigen gene (staphylococcal enterotoxin B, SEB) as immune-adjuvant plus the immunostimulatory gene canine GM-CSF, the objective responses and median survivals for spontaneous canine melanoma were as follows: stage I, 3/3-427 days; stage II, 3/5-399 days; stage III, 4/12-168 days; stage IV, 0/2-n.d. Median survival (168 days) of stage III patients (*n* = 12) resulted significantly higher than historical surgery controls (105 days) [14]. Intratumoral expression of SEB and canine GM-CSF genes carrying plasmid did not induce clinically significant toxicity in treated dogs.

A totally different approach to promote immunologic “priming” through induction of apoptosis was assayed for canine melanoma [15]. Direct intratumoral injections of a plasmid carrying the human Fas ligand (hFasL) gene caused a quick reduction (7 days) of tumor burden that was seen in 3 of 5 treated dogs. At that time, each dog was provided with standard care therapy as indicated for each tumor (surgery, radiation, or palliation), and the final result was 4 objective responses with survivals ranging from 168 to 574 days. The treatment did not show any adverse effect.

On the other hand, intramuscular needle free jet injection of a plasmid containing human tyrosinase gene and its expression as a melanoma xenoantigen considerably prolonged the median survival of canine melanoma bearing patients up to 389 days as compared with historical controls subjected to standard treatments (60–150 days) [16]. In addition, at least two long-term survivors displayed detectable levels of humoral anti-human tyrosinase antibodies [17]. Fifty-eight patients were enrolled in a larger trial to evaluate the safety and efficacy of this DNA vaccine as adjunctive treatment for oral malignant canine melanoma in which locoregional disease control was achieved [18]. While both safety and efficacy were confirmed, the median survival due to melanoma was significantly higher (>750 days) as compared to historical controls (324 days) and reported only 14/58 deaths were due to melanoma compared to 34/53 in reported historical controls [19]. In a further trial, hTyr and other xenogeneic DNAs coding for murine tyrosinase (mTyr), murine gp75, and human GM-CSF were assayed at increasing doses. Combinations of mTyr and hGM-CSF were also evaluated [20]. Thirty-three stage II-III dogs with locoregionally controlled canine melanoma across the xenogeneic vaccine studies displayed a median survival time of 569 days, largely exceeding the value expected for standard treatments. Minimal to mild pain was noted at vaccination sites. All these results served to support the approval of the first therapeutic cancer vaccine available in the veterinary pharmaceutical market. As a continuation of these studies, a new one determined the safety and effectiveness of the murine tyrosinase xenogeneic vaccine for canine digit melanoma when used in conjunction with local and regional disease control [21]. This retrospective study suggests a prolongation of survival for dogs with melanoma of the digit treated with xenogeneic DNA melanoma vaccine (a median survival time: 476 days) compared with historical controls treated with surgery alone (a median survival time: 365 days). A survival advantage was noted for those dogs that were vaccinated near the time of diagnosis over those dogs that had a significant delay between diagnosis and vaccination.

An independent retrospective study of 22 hTyr vaccinated versus 23 nonvaccinated melanoma-bearing dogs did not show significant differences in progression-free survival, disease-free interval, or median survival time [22]. This new outcome suggests the need of new controlled trials with a larger amount of patients.

Autologous dendritic cells pulsed with adenoviral vector encoding human gp100 were successfully produced and applied as radiotherapy adjuvant to treat canine melanoma [23]. The 3 treated patients exceeded the expected median survival (210–1440 days), even though one died sooner because of progressive disease. No adverse effects were observed in any of the treated dogs with either the priming or the subsequent vaccine booster doses.

In a different approach involving vaccination with allogeneic melanoma cells expressing xenogeneic human gp100, melanoma bearing dogs experiencing tumor control survived significantly longer than dogs having no response (median survivals: 337 days versus 95 days) [24]. Adverse reactions were limited to mild induration and erythema at the site of vaccination.

A feasibility study was performed for adenovector CD40 ligand (AdCD40L) immunogene treatment [25]. One case of advanced stage III oral melanoma, treated by intratumoral vaccination followed by cytoreductive surgery, did not relapse and survived for 401 days. A second case of conjunctival malignant melanoma, treated only with intralesional injections, showed a continuous remission for more than 150 days. Only reversible minor side effects were reported. Following this research, a pilot study to treat canine melanoma (14 oral, 4 cutaneous, and 1 conjunctival) with local AdCD40L was reported [26]. One to 6 intratumoral injections of AdCD40L were given every 7 days, followed by cytoreductive surgery in 9 cases and only immunotherapy in 10 cases. Posttreatment Immune stimulation was evidenced by tumor tissue infiltration with T and B lymphocyte. The best overall response included 5 complete responses, 8 partial responses, and 4 stable and 2 progressive diseases. Median survival was 160 days (range, 20–1141 days), with 3 dogs still alive at submission. This work demonstrated that local AdCD40L therapy was safe and could have beneficial effects on dogs.

The immunogenicity, safety, and therapeutic efficacy of a human chondroitin sulfate proteoglycan-4 (hCSPG4) DNA-based vaccine were evaluated [27]. Dogs with stage II-III surgically resected CSPG4-positive oral malignant melanoma were subjected to monthly intramuscular plasmid administration, which was followed immediately by electroporation (electrovaccination) from 6 to 20 months. Overall (653 versus 220 days) and disease-free (477 versus 180 days) survival times were significantly longer in 14 vaccinated dogs as compared with 13 nonvaccinated controls. No clinically relevant local or systemic side effects were found. This suggested that xenogeneic electrovaccination against CSPG4 is able to overcome host unresponsiveness to the “self” antigen and seems to be effective in treating canine malignant melanoma.

By adding local* herpes simplex* virus thymidine kinase (HSV-*tk*) suicide gene bearing plasmid plus ganciclovir, in an approach that included intratumoral injections of xenogeneic hamster cells secreting hIL-2 and hGM-CSF (*n* = 45), melanoma bearing dogs presenting local objective responses survived significantly longer than dogs lacking local objective responses (220 days versus 151 days), being both responses considerably higher than those of surgery controls (82 days) [28].

As a continuation of these encouraging data, a surgery adjuvant treatment against the minimal residual disease whose rationale appeared to be considerably superior to the previous intratumoral treatment was assayed [29, 30]. This new treatment consisted of complete or cytoreductive surgery followed by a combination of suicide gene therapy with a subcutaneous vaccine. This vaccine was a mixture of formolized tumor cells and irradiated xenogeneic cells producing hIL-2 and hGM-CSF. The postsurgical margin of the cavity was infiltrated with lipid-complexed HSV-*tk* suicide gene coadministrated with ganciclovir. Toxicity was absent or minimal in all patients. With respect to surgery-treated controls (ST), the complete surgery (CS) arm of this combined treatment (CT) significantly increased the fraction of local disease-free patients from 13 to 81% and distant metastases free from 32 to 84%. Even though it is less effective than the CS arm, the partial surgery (PS) arm of this CT was significantly better controlling the disease than only surgery (14% while PS-ST: 0% and CS-ST: 5%). In addition, CT produced a significant sevenfold (CS) and threefold (PS) increase in overall survival. The CS-CT arm significantly improved both CS-ST metastasis-free and melanoma overall survival from 99 days (respective ranges: 11–563 and 10–568) to >2848 days (81–2848 and 35–2848). Thus, more of 50% of CT patients died of melanoma unrelated causes, transforming a lethal disease into a chronic one. After an extensive follow-up (9 years) with a high number of treated patients (*n* = 283), this study suggested that the most optimal clinical setting is this surgery adjuvant treatment against minimal residual disease.

To overcome the limitations imposed by the costly production and delivery of cytokine producing transgenic xenogeneic cells as well as the appearance of canine melanoma resistant to suicide gene therapy, we designed a new trial where these cells were replaced by lipoplexes carrying the corresponding cytokines genes in the subcutaneous vaccine and canine interferon-*β* (cIFN-*β*) strengthened the local antitumor effects [31]. After 6 years of follow-up, 301 canine patients were subjected to the combined treatment as surgery adjuvant and 162 remained as surgery controls. While maintaining a similar efficacy and safety profile with respect to the previous trial [30], patients subjected to partial surgery followed by the combined treatment displayed significantly longer overall survivals.

In summary, the strategy combining local antitumor gene therapy together with a systemic vaccine enhanced by cytokines not only delayed or prevented recurrence and distant metastasis, but also substantially extended disease-free and overall survival, with a consequent improvement in the quality of life.

## 3. Canine Soft Tissue Sarcoma

Soft tissue sarcomas involve a group of tumors with differing morphological features that share similar biological behaviors. These tumors arise from many nonbony connective tissues and may originate in visceral and nonvisceral sites, comprising approximately 15% of all skin and subcutaneous tumors in the dog [32]. Wide surgical excision remains the cornerstone of treatment for these tumors. Local recurrence is common following conservative resection, and recurrent tumors are more difficult to treat.

A superantigen gene was assayed to enhance the action of immunostimulant cytokine gene, in this case against the invasive spontaneous canine soft tissue sarcoma that is refractory to most conventional therapies other than surgical excision. So the effects of intralesional treatment with a plasmid containing bacterial superantigen gene (staphylococcal enterotoxin A, SEA) plus immunostimulatory canine IL-2 gene before surgical resection or exploration and biopsy of the tumor bed were reported [33]. Adverse effects related to treatment were transient and manageable, and the median survival of the responders was >540 days without experiencing local recurrence or metastasis.

A polygene therapy approach was also assayed by our unit [34]. Eleven soft tissue sarcoma canine patients were subjected to (i) periodic subcutaneous injection of irradiated xenogeneic cells secreting hGM-CSF and hIL-2 mixed with allogeneic or autologous tumor homogenates; and (ii) injections of cIFN-*β* and HSV-*tk*-carrying lipoplexes and ganciclovir, marginally (after surgery), and/or intratumorally (in the case of partial tumor resection, local relapse, or small surface tumors). This treatment alone or as surgery adjuvant was safe and well tolerated. In those patients presenting local disease (6/11), the suicide gene plus cIFN-*β* treatment induced local antitumor activity evidenced by the objective response (3 complete, 1 partial) and stable disease (2). In addition, the treatment prevented or delayed local relapse, regional metastases (lymph nodes developed only in 1/11), and distant metastases (0/11), suggesting a strong systemic antitumor immunity. Most of the patients displayed long survival times while maintaining a good quality of life: 2 about 4 years, 2 about 3 years, 1 more than 2 years, 4 more than 1 year, and 2 more than 6 months.

## 4. Canine Osteosarcoma

Osteosarcoma accounts for approximately 85% of primary bone cancers in the dog [35]. It is a common cancer of large to giant breed dogs, and it occurs primarily in the appendicular skeleton. Osteosarcoma in dogs is a common and highly metastatic tumor that biologically closely resembles osteosarcoma in humans [4, 5] and does not have additional treatment options when adjuvant chemotherapy has failed against its disseminated form [36]. Even with removal of the primary tumor before spread of the cancer is clinically detectable, metastases to lung, bone, or other sites eventually develop in almost all dogs [35].

Metastatic disease often needs a systemic delivery of the therapeutic molecule to reach multiple points simultaneously. Systemic gene delivery by intravenous injection of cationic liposome-DNA complexes carrying canine IL-2 gene was assayed in 22 dogs with chemotherapy-resistant osteosarcoma lung metastases [36]. The treatment was well tolerated with 3 dogs displaying medium-term objective responses and 4 cases of stable disease. A significant increase of the median survival (82 d) with respect to untreated controls (58 d) was observed.

As it was the case for soft tissue sarcomas [34], 6 osteosarcoma bearing patients were subjected to a treatment combining (i) the local antiproliferative effects of cIFN-*β* and HSV-*tk* suicide gene therapy with (ii) the systemic effects triggered by osteosarcoma antigens in an immunostimulatory environment created by the slow secretion of hGM-CSF and hIL-2 [37]. Beyond the high safety standard of the proposed treatment on all the patients, 4 of them survived more than 6 months (among them, two exceeded 1 year). In addition, the treatment prevented or delayed local relapse, regional metastases, and distant metastases, suggesting a strong systemic antitumor immunity. The use of this treatment after surgical removal of the tumor was safe and could delay or prevent postsurgical recurrence and metastases, with the consequent quality of life and survival rate improvement. In addition, when diagnosed at the early stages, a peritumoral application of cIFN-*β* plus suicide genes in combination with subcutaneous vaccine could be effective in controlling both local (recurrence) and distant disease (metastases), as evidenced by one case of long-lasting complete remission (last update: >453 days) without surgical intervention.

More recently, intratumoral FasL gene was delivered before surgery in an adenovirus vector (Ad-FasL) as neoadjuvant to standard of care in 56 canine osteosarcoma patients [38]. Tumors from treated dogs had greater inflammation, necrosis, apoptosis, and fibrosis compared to the pretreatment condition or no treated dogs. Survival correlated with the degree of inflammation or lymphocyte-infiltration scores, as well as apoptosis scores.

## 5. Canine Lymphoma

Lymphoma represents the most frequent hematopoietic cancer in dogs and often presents in advanced stage (III–V) at diagnosis and, most commonly, has an aggressive clinical course requiring prompt treatment. However, although complete remission may be achieved using multiagent chemotherapy, the mortality rate from this neoplasm remains high [39].

The use of hGM-CSF secreting autologous tumor cell vaccine was safe, but it did not result in clinical benefit for canine B-cell lymphoma patients [40].

In another* ex vivo* approach, Emm55, a* Streptococcus pyogenes* serotyping antigen gene, was transferred to autologous tumor cells of 7 canine lymphoma patients [41]. Once treated, all of them developed an antibody response to multiple autologous tumor antigens and CD8+ mediated cellular cytotoxicity. One long-lasting complete response (>18 months) and 3 extended survivals support further trials. No adverse effects related to treatment were informed.

A vaccine against dog telomerase reverse transcriptase (dTERT) transferred by adenoviral vector electroporation was also proposed [42] as a valid target for immunotherapy of malignant lymphoma when combined with standard chemotherapy regimen. dTERT-specific immune response was induced in 13 out of 14 treated animals (93%) and remained detectable and long-lasting with the absence of autoimmunity or other side effects. Most interestingly, the survival time of vaccine/chemotreated dogs was significantly increased over historic controls of chemotreated animals (>97.8 versus 37 weeks). Following this study, a double-arm clinical trial with an extended number of B-cell lymphoma patients was conducted [43], to measure the antigen-specific immune response and to evaluate the potential toxic effects of immunotherapy during 3.5 years of follow-up. Without adverse effects, the overall survival time of vaccine/chemotherapy-treated dogs was significantly increased over the chemotherapy-only group (>76.1 versus 29.3 weeks) and dTERT expression levels in tumor cells correlated with overall survival among vaccinated patients.

Based on a RNA mediated gene therapy, a completely different way was proposed to treat non-Hodgkin's lymphoma [44]. In that case, when administered to dogs in remission after induction chemotherapy,* ex vivo* autologous tumor RNA electroporated CD40-activated B cells safely stimulated immunity* in vivo*. This approach was safe and potentiated the effects of salvage therapy, improving the rate of durable second remissions as well as subsequent lymphoma-specific survival following salvage therapy.

## 6. Other Canine Tumors

Electrochemogene therapy (ECGT) combining electroporation with a feline interleukin-12 (fIL-12) carrying plasmid and bleomycin was very effective [45]. It was able to control two oral squamous cell carcinomas and one acanthomatous ameloblastoma tumor that displayed long-lasting complete responses (56, 27, and 9 months tumor-free, resp.).

An astrocytoma bearing patient was treated by a combination of surgery, intracavitary adenoviral human interferon-*γ* (IFN-*γ*) gene transfer, and vaccination with glioma cell lysates mixed with CpG oligodeoxynucleotides [46]. Transient neurological symptoms were resolved and this dog remained tumor-free over 450 days following surgery.

Intramuscular electrogene therapy (EGT) with plasmid encoding human interleukin-12 (hIL-12) was assayed in dogs with spontaneously occurring tumors, being a feasible and safe procedure. The systemic transgene expression of hIL-12 induced endogenous canine IFN-*γ* release and tumor growth control in 4 of 6 treated dogs (complete response for two mast cell tumors and stable disease for one pulmonary histiocytic sarcoma and one osteosarcoma) [47].

Malignant cutaneous canine mast cell tumors were treated with EGT using DNA plasmid encoding hIL-12 [48]. This treatment produced a significant reduction of treated tumors' size, ranging from 13% to 83% (median 50%) of the initial tumor volume (*n* = 8). There were histological changes and a reduction in number of malignant mast cells, as well as an inflammatory cell infiltration of treated tumors. No local or systemic adverse side effects were detected.

## 7. Feline Fibrosarcoma

Fibrosarcoma is a deadly disease in cats and is significantly more often located at classical vaccine injections sites. Due to poor cure rates with surgery alone, the additional use of adjuvant radiation therapy and/or chemotherapy has been under investigation at multiple veterinary cancer centers for the last few years [49].

The pioneering work of veterinary cancer gene therapy [2] also described the effects of the* ex vivo* genetically engineered xenogeneic cell gene therapy approach on the highly invasive feline fibrosarcoma that often presents postsurgical local relapse. There, they evaluated the effects of repeated peritumoral local injections of human IL-2 secreting cells after surgical removal of the tumor followed by ^192^Ir-brachytherapy. This combined treatment resulted in a significantly longer median survival of > 480 days compared to 240 days of the surgery plus radiation controls (both groups: *n* = 16). Interestingly the combined treatment drastically reduced the relapse rate after 480 days of treatment from 69% in the control group to 31%.

In a different approach, the peritumoral injection of feline IL-2 or human IL-2 genes carried by poxviral vectors was assayed as adjunct treatment following surgery and ^192^Ir-brachytherapy for feline fibrosarcoma [50]. The treatment significantly diminished the rate of recurrence at day 365 from 11/18 (control) to 5/18 (feline IL-2) and 7/18 (human IL-2).

A feasibility study combined fractionated radiotherapy, hyperthermia, and heat-inducible intratumor adenoviral fIL-12 gene therapy for feline soft tissue sarcoma determining the dose-dependent systemic toxicity in phase I trial [51].

Two reports from the same research team used magnetofection for intratumoral gene delivery of feline fibrosarcoma. The first one was phase I dose-escalation study to determine a safe dose [52]. Twenty-five client-owned cats with clinical diagnosis of fibrosarcoma—primary tumors as well as recurrences—entered the study. Four increasing doses of plasmids coding for feline cytokines fIL-2, fIFN-*γ*, or fGM-CSF (from 15 to 450 *μ*g each) were assayed. Two preoperative intratumoral injections of the magnetic DNA solution were followed by magnetofection. With only 1 of 25 treated cats showing adverse events at the highest dose, the treatment was considered to be safe. Altogether 6 cats developed local recurrences during a 1-year observation period. A second phase I dose-escalation study was performed to determine toxicity and feasibility of gene therapy with fGM-CSF coding plasmid attached to magnetic nanoparticles (from 50 to 1250 *μ*g) in 20 cats with fibrosarcomas [53]. Two preoperative intratumoral injections followed by magnetofection were given. Without significant treatment related toxicity, 10 of 20 treated cats were recurrence-free on day 360.

## 8. Equine Melanoma

Melanomas are among the most common skin tumors in horses, with prevalence rates reaching as high as 80% in adult gray horses. Most melanocytic tumors are benign at initial presentation; however, if left untreated, up to two-thirds can progress to overt malignant behavior and there are limited therapeutic options in metastatic disease [54].

Based on our experience with spontaneous canine melanoma, we assayed the combination of local suicide gene therapy with a cancer vaccine following surgical removal of the superficial tumors in a single male gray horse patient with metastatic melanoma [55]. The patient presented multiple grouped superficial perianal and foreskin lesions that were surgically removed. Some isolated subcutaneous masses in the neck, back, and shoulders that were injected with the suicide gene plus prodrug (HSV-*tk* gene carrying plasmid, ganciclovir). Excised tumors were used to prepare a vaccine that was subcutaneously injected in the flanks as scheduled for canine treatment [29]. No undesirable side effects were observed. After a six-month lasting treatment, the patient that survived more than 2 years recovered its quality of life including its full reproductive function, without local relapse at the surgery areas.

As far as we know there are only three additional reports on equine cancer gene therapy. In the first one [56] melanoma metastases in gray horses were treated with a plasmid encoding immunostimulatory hIL-12 (*n* = 7). A mean reduction in tumor size to 41% was observed after one cycle, compared to 88% in the group treated with noncoding control vector and 107% in untreated animals. This transient volume reduction could be repeated with subsequent hIL-12 plasmid transfer. No side effects of the treatment were observed. In the second trial, a randomized double-blind, placebo-controlled study was conducted to investigate the antitumor effects of hIL-18- or hIL-12-encoding plasmid DNAs, intratumorally injected in gray horses with metastatic melanoma [57]. Significant tumor regression could be shown in both cytokine gene treated groups whereas placebo-treated control patients showed tumor growth. In addition, 7 of 10 tumors from horses treated with hIL-18 or hIL-12 showed peritumoral and/or intratumoral inflammatory infiltrates after treatment compared with 1 of the 6 in the control group. The treatment was safe and well tolerated.

In a parallel study [58], 7 grey horses bearing melanoma were intratumorally injected with 250 *μ*g of naked plasmid DNA coding for equine IL-12 (eIL-12). More than 99% of the plasmid disappeared within 36 hours. The variable quick increase of IFN-*γ* expression after eIL-12 plasmid injection indicated biological activity. Intratumoral injection of plasmid DNA was a feasible method for inducing transgene expression* in vivo*.

## 9. Conclusion

Most of the veterinary cancer gene therapy trials on patients with spontaneous tumors could be classified as immunogene therapy and were performed with nonviral vectors. In general terms very slight or no adverse collateral effects were found and most of the times treated animals displayed a better course of the disease (longer survival, delayed or suppressed local or systemic relapse, and recovery of the quality of life) suggesting the utility of this sort of strategies as standard adjuvant treatments. Besides, some of these trials also demonstrated the utility of spontaneous tumors in companion animals as a valid translational model for the evaluation of novel DNA based therapies.

Two emerging approaches were supported by a large number of treated patients: the xenogeneic vaccine [16] and the suicide gene plus autologous/allogeneic vaccine [29, 30]. Both systems based the systemic disease control on the immune system following the surgical removal of the tumor. While the first approach using hTyr vaccine was specific for melanoma [18], the second approach appeared to be more versatile, because it was successfully translated to other kinds of tumors such as soft tissue sarcoma [34] and osteosarcoma [37].

As it is the case for human patients, usually survival times for those veterinary patients that displayed objective responses were significantly higher than for nonresponders. In addition better results were obtained when treating the early stages of the disease and, in the case of advanced disease presenting bulky tumors, previous standard procedures such as surgery, chemotherapy, and/or radiotherapy were necessary to obtain the best outcome of the whole treatment.

It is worth to note that the efficacy displayed by plasmid-based nonviral vectors used in 80% of the trials involving large animals demonstrated their usefulness for present and future gene therapy treatments. On the other hand, new developments in oncolytic adenoviral vectors were recently reported [59] and they could be eventually combined with immunogene therapy approaches. Because of safety issues and the costs of vector production, nonviral vectors are more likely to be sooner approved and available for the treatment of veterinary oncologic patients.

Although intravenous injections of cationic lipid: DNA complexes had some immune mediated antitumor activity against canine soft tissue sarcoma regardless the transgene carried by the plasmid in the case of angiostatin “therapeutic” or luciferase “reporter” gene [60], this fact should not be taken as a negative result. In addition to the immune enhancing effects of complexes carrying noncoding plasmid in a vaccine proposed against hemangiosarcoma [61], the expression of therapeutic genes displayed specific antitumor activity as repeatedly evidenced in many papers listed in Tables 1, 2, and 3. Further work has to be done to ascertain the contribution of each component* in vivo*.

To avoid local recurrence, cancer control requires not only converting the immune tumor environment from tolerant to competent, but also waking a systemic immune response against the metastatic spread. Being cancers complex diseases that involve multiple interactions among many cell types beyond tumor and immune cells, the most effective cancer therapies may consist of combinations of diverse immunogene therapy strategies with other cytotoxic gene therapies as well as rational combinations with other standard therapies such as surgery, radiotherapy, targeted molecular therapies, and conventional chemotherapy. As displayed in Tables 1 and 2, most of canine and feline immunogene therapy trials were combined with the above-mentioned standard therapies.

An increasing interest in cancer therapy trials in companion animals (especially dogs and cats) including those using gene therapy techniques will serve as concept, safety, and effectiveness proofs. Despite the complexity for developing multicenter consortia capable of conducting clinical studies in advance or in parallel with human clinical trials, this new trend in the research of novel cancer therapies would indeed benefit both companion animals and human patients [5, 62–64].

Trials in companion animals are shorter in time and at lower costs than in humans. Nowadays, there are conditions for the development of translational research in the oncology field [65, 66], intending to apply scientific information to solve present problems in relatively brief periods of time. Proper cancer models involving companion animals spontaneously induce the investigators to work in the duality between the veterinary perspective and the potential application to human medicine. However, these are not contradictory objectives, and more trials in humans based on the equivalent trials in pets will possibly come soon.

## Figures and Tables

**Table 1:** Canine cancer immunogene therapy trials.

						
#	Genes	Tumor	Vector	Mode	Results	Authors/year/references
	Cytokines	Canine				
1	hIL-2	MEL	Irradiated hIL-2 producing xenogeneic cells made by plasmid transfection	*Ex vivo/*p.t./ +SX + RX	(*n* = 16) NR, 480 d. Control: 1/treated: 6. Median survival: treated: 270 d/control: 75 d	Quintin-Colonna et al., 1996 [2]
2	cIL-2 + enterotoxin-A	STS	Plasmid lipofection	*In vivo*/i.t. + SX	(*n* = 16) 3 CR, 1 PR, 1 SD (84 d). Responders survival: >540 d w/o relapse.	Thamm et al., 2003 [33]
3	cIL-2	OSA lung metastases	Plasmid lipofection	*In vivo/*i.v./ +SX + CHT	(*n* = 20) 84 d: 1 CR, 2 PR, 4 SD. Median survival (s IV): treated: 82 d/Hist. Surg. controls: 58 d	Dow et al., 2005 [36]
4	hGM-CSF	MEL FSA OSA HGS	Irradiated hGM-CSF producing autologous tumor cells plasmid transferred by gene gun	*Ex vivo/*i.d./ NAT	MEL (*n* = 10) 1 CR (300 d), 1 PR (300 d), 1 NED (250 d), 1 SD (125 d) FSA (*n* = 3) 1 PR, 1 NED (175 d)	Hogge et al., 1998 [13]
5	cGM-CSF + enterotoxin-B	MEL	Plasmid lipofection	*In vivo/*i.t., p.t., i.l.n./NAT	(*n* = 12) median survival (stage III): treated: 168 d/Hist. Surgery controls: 105 d	Dow et al., 1998 [14]
6	hGM-CSF	LYP	Plasmid lipofection	*In vivo/*i.d./ +CHT	Vaccine (*n* = 26)/placebo (*n* = 26). Median survival 474 d/342 d	Turek et al., 2007 [40]
7	hIL-2 + hGM-CSF HSV-*tk *	MEL	Plasmid lipofection: HSV-*tk* + GCV. Irradiated xenogeneic hIL-2 and hGM-CSF producing cells made by plasmid lipofection	*In vivo* (SG)/i.t.,* Ex vivo *(CPXC)/i.t./NAT	Combined treatment (*n* = 45)/surgery controls (*n* = 23). Median survival: 160 d/82 d. Metastasis-free median survival: >509 d/133 d. Combined treatment: CR = 16%, PR = 31%, SD = 27%, PD = 27%.	Finocchiaro et al., 2008 [28]
8	hIL-2 + hGM-CSF HSV-*tk *	MEL	Plasmid lipofection: HSV-*tk* + GCV. Irradiated xenogeneic hIL-2 and hGM-CSF producing cells made by plasmid lipofection	*In vivo *(SG) − i.t*./Ex vivo *(CPXC) + (TV) − s.c./+SX	Combined treatment (*n* = 100)/surgery controls (*n* = 51): local disease-free patients: 58%/6%. Metastasis-free patients: 78%/43%. Median survival: >1312 d/78 d. Metastasis-free median survival: >1312 d/112 d.	Finocchiaro and Glikin, 2008 [29]
9	hIL-2 + hGM-CSF HSV-*tk *	MEL	Plasmid lipofection: HSV-*tk* + GCV. Irradiated xenogeneic hIL-2 and hGM-CSF producing cells made by plasmid lipofection	*In vivo *(SG) − i.t. *Ex vivo *(CPXC) + (TV) − s.c./+SX	Combined treatment (*n* = 283)/surgery controls (*n* = 135). Complete surgery arms: local disease-free patients: 81%/13%. Metastasis-free patients: 84%/32%. Median survival: >2848 d/99 d. Metastasis-free median survival: >2848 d/99 d.	Finocchiaro and Glikin, 2012 [30]
10	hIL-2 + hGM-CSF cIFN-*β* + HSV-*tk *	MEL	Plasmid lipofection: HSV-*tk* + IFN-*β* + GCV. Plasmid lipofection: hIL-2 and hGM-CSF	*In vivo *(SG) + (IFN-*β*) − i.t. *In vivo* (hIL-2 + hGM-CSF) + (TV) − s.c./+SX	Combined treatment (*n* = 301)/surgery controls (*n* = 162). Complete surgery arms: local disease-free patients: 83%/11%. Metastasis-free patients: 89%/44%. Median survival: >2211 d/109 d. Metastasis-free median survival: >2211 d/134 d.	Finocchiaro et al., Submitted [31]

	Genes Cytokines	Tumor Canine				

11	hIL-2 + hGM-CSF cIFN-*β* + HSV-*tk *	STS	Plasmid lipofection: HSV-*tk* + IFN-*β* + GCV. Irradiated xenogeneic hIL-2 and hGM-CSF producing cells made by plasmid lipofection	*In vivo* (SG) + (IFN-*β*) − i.t. *Ex vivo* (CPXC) + (TV) − s.c/+SX.	(*n* = 11) 5 survived >2 years, 4 survived >1 year	Finocchiaro et al., 2011 [34]
12	hIL-2 + hGM-CSF cIFN-*β* + HSV-*tk *	OSA	Plasmid lipofection: HSV-*tk* + IFN-*β* + GCV. Irradiated xenogeneic hIL-2 and hGM-CSF producing cells made by plasmid lipofection	*In vivo* (SG) + (IFN-*β*) − i.t. *Ex vivo* (CPXC) + (TV) − s.c/+SX.	(*n* = 6) 2 survived > 1 year	Finocchiaro et al., 2012 [37]
13	hIFN-*γ*	ASC	Adenovirus	*In vivo* − p.t. + (TV) − s.c./ +SX.	(*n* = 1) feasible. Disease-free survival >450 d	Pluhar et al., 2010 [46]
14	fIL-12 + bleomycin	SCC AMB MEL STS	Naked plasmid + bleomycin electrotransfer	*In vivo* − i.t./+CHT	(*n* = 5) 2 SSC and AMB resolved (56, 27, 9 months tumor-free).	Reed et al., 2010 [45]
15	hIL-12	MCT PHS OSA MAC	Naked plasmid electrotransfer	*In vivo* − i.m/ ±SX ± CHT	(*n* = 6) CR: 2 of 3 MCT. SD: 1 PHS and 1 OSA.	Cemazar et al., 2011 [47]
16	hIL-12	MCT	Naked plasmid electrotransfer	*In vivo* − i.t./ ±SX ± CHT	(*n* = 8) 50% median reduction of tumor volumes	Pavlin et al., 2011 [48]

	Antigens	Canine				

17	hTyr	MEL oral	Naked plasmid − Jet injection	*In vivo* − i.m./ ±SX ± RX	(*n* = 9) 1 CR, 2 NED, 1 SD (14 d)/median survival: 389 d. Historical controls (s II 150 d, s III 60 d). Humoral antibodies: 3/9.	Bergman et al., 2003 [16] Liao et al., 2006 [17]
18	hTyr/mTyr mgp75/ hGM-CSF	MEL	Naked plasmid − Jet injection	*In vivo* − i.m./ ±SX ± RX	Different combinations and doses. Median survival stages II-III (*n* = 33) w/locoregional control: 569 d. 25/33 still alive at submission.	Bergman et al., 2006 [20]
19	hTyr	MEL oral	Naked plasmid − Jet injection	*In vivo* − i.m./ +SX ± RX	(*n* = 58) death attributable to MM (*n* = 15), to other causes (*n* = 16). Median survival: >750 d. Historical controls (*n* = 53): 324 d.	Grosenbaugh et al., 2011 [18]
20	mTyr	MEL digital	Naked plasmid − Jet injection	*In vivo* − i.m./ +SX + RX	(*n* = 58) median survival: >476 d. Surgery only historical controls: 365 d	Manley et al., 2011 [21]

	Antigens	Canine				

21	hTyr	MEL	Naked plasmid − Jet injection	*In vivo* − i.m/ +SX ± RX	(*n* = 22) no differences in PFS, DFI, or median survival with surgery controls (*n* = 23)	Ottnod et al., 2013 [22]
22	hgp100	MEL	Autologous adenovirus transduced dendritic cells	*Ex vivo* − s.c./ +SX + RX	(*n* = 3) survival ranging 210–1440 d	Gyorffy et al., 2005 [23]
23	hgp100	MEL	Irradiated allogeneic hgp100 expressing tumor cells plasmid transferred by gene gun	*Ex vivo* − i.d./ NAT	(*n* = 34) median survival: responders (CR, PR, SD) (*n* = 12), 337 d/nonresponders (*n* = 20), 95 d	Alexander et al., 2006 [24]
24	cTERT cTERT-LTB	LYP	Naked plasmid electroporation + adenovirus 6	*In vivo* − i.m./ +CHT	(*n* = 14) GT + *CHT*/(*n* = 8) *CHT*. Median survival: >96 wks/28 wks. Time to first relapse: >26 wks/12 wks.	Peruzzi et al., 2010 [42]
25	cTERT	LYP	Electroporation + Adenovirus	*In vivo* − i.m./ +CHT	(*n* = 21) VAC/(*n* = 21) CTR median survival: VAC 76.1 w/CTR 29.3 w	Gavazza et al., 2013 [43]
26	Emm55	LYP	Plasmid/electroporation	*Ex vivo* − i.v./ ±CHT	(*n* = 7) 1 CR > 555 d, 6 PR, 3 extended survival >2X	Lawman et al., 2008 [41]
27	CSPG4	MEL	Plasmid/electroporation	*In vivo* − i.m./. +SX.	(*n* = 14) VAC/(*n* = 13) CTR median survival: VAC 76.1 w/CTR 29.3 w	Riccardo et al., 2014 [27]
28	cNHL-mRNA	LYP	Combination chemotherapy + NHL-mRNA loaded electroporation of CD40-activated B cells.	*Ex vivo* RNA transfer/ +CHT	(*n* = 19) significant increase of lymphoma-specific survival following salvage therapy	Sorenmo et al., 2011 [44]

	Specific ligands	Canine				

29	hCD40L	MEL	Adenovirus	*In vivo* − i.t./. ±SX.	(*n* = 2) feasible and manageable toxicity 1 NED 401 d 1 PR >150 d	von Euler et al., 2008 [25]
30	hCD40L	MEL	Adenovirus	*In vivo* − i.t./. ±SX.	(*n* = 19) median survival: responders (CR, PR, SD) (*n* = 17), 160 d/nonresponders (*n* = 2)	Westberg et al., 2013 [26]
31	hFasL	MEL	Plasmid lipofection	*In vivo* − i.t./ ±SX ± RX	(*n* = 5) GT: 3 TR, 1 SD/CT: 2 CR, 2 PR Survival: 168–574 d	Bianco et al., 2003 [15]
32	hFasL	OSA	Adenovirus	*In vivo* − i.t./ +SX	(*n* = 48) Ad-FasL-mediated tumor inflammation improved median survival (score 2/3: 359 d; controls: 221 d)	Modiano et al., 2012 [38]

AMB, acanthomatous ameloblastoma; ASC, astrocytoma; FSA, fibrosarcoma; HGS, hemangiosarcoma; LYP, lymphoma; MAC, mammary adenocarcinoma; MCT, mast cell tumor; MEL: melanoma; OSA, osteosarcoma; PHS, pulmonary histiocytic sarcoma; SCC, squamous cell carcinoma; STS, soft tissue sarcoma; CD40L, CD40 ligand; Emm55, *Streptococcus pyogenes* serotyping antigen; FasL, Fas ligand; GM-CSF, granulocyte macrophage colony stimulating factor; gp100, glycoprotein 100; gp75, glycoprotein 75; HSV-*tk*, herpes simplex thymidine kinase; IFN-*β*, interferon-*β*; IFN-*γ*, interferon-*γ*; IL-12, interleukin-12; IL-2, interleukin-2; LTB, *Escherichia coli* heat labile enterotoxin; NHL-mRNA, non-Hodgkin lymphoma messenger RNA; TERT, telomerase reverse transcriptase; Tyr, tyrosinase; CR, complete response; CTR, control; DFI, disease-free interval; GT, gene therapy; NED, no evidence of disease; NR, no relapse; PFS, progression-free survival; PR, partial response; SD, stable disease; CPXC, cytokine producing xenogeneic cells; GCV, ganciclovir; SG, suicide gene; TV, tumor vaccine; VAC, vaccinated; i.d., intradermal; i.l.n., intralymph node; i.m., intramuscular, i.t., intratumoral; i.v., intravenous; p.t., peritumoral; s.c., subcutaneous; CHT, chemotherapy; NAT, no additional treatment during or following gene therapy; RX, radiotherapy; SX, surgical excision; d, days; wks, weeks; prefixes: c, canine; e, equine; f, feline; h, human; m, murine.

**Table 2:** Feline cancer immunogene therapy trials.

#	Genes	Tumor	Vector	Mode	Results	Authors/year/reference
	Cytokines					
1	hIL-2	FSA	Irradiated xenogeneic hIL-2 producing cells made by plasmid transfection	*Ex vivo/*p.t./ +SX + RX	(*n* = 16) no relapse at 480 d: 5. Control/11. Treated: 11. Median survival: control: 240 d/treated: >480 d	Quintin-Colonna et al., 1996 [2]
2	hIL-2/fIL-2	FSA	Poxviruses	*In vivo* − i.t./ +SX + RX	(*n* = 18) no relapse at 365 d: control: 7/treated: 13 fIL-2:/11 hIL-2	Jourdier et al., 2003 [50]
3	fIL-12	FSA	Adenovirus	*In vivo* − i.t./ +RX + HT	(*n* = 13) feasible and manageable toxicity. Maximally tolerated safe dose: 10^10^ pfu	Siddiqui et al., 2007 [51]
4	fIL-2 + fIFN-*γ* + fGM-CSF	FSA	Iron oxide PEI-complexed plasmid enhanced by magnetofection	*In vivo* − i.t./ +SX	(*n* = 21) dose escalation and safety. Maximal dose of plasmids (450 *μ*g each) was well tolerated. Maximal effects expected at 150 *μ*g	Jahnke et al., 2007 [52]
5	fGM-CSF	FSA	Iron oxide PEI-complexed plasmid enhanced by magnetofection	*In vivo* − i.t./ +SX	(*n* = 20) (*n* = 25) dose escalation and safety. Maximal dose of plasmid (1250 *μ*g) was well tolerated. 10 recurrence free after 360 d.	Hüttinger et al., 2008 [53]

hIL-2, human interleukin-2; fIL-12, feline interleukin-12; fIL-2, feline interleukin-2; fIFN-*γ*, feline interferon-*γ*; fGM-CSF, feline granulocyte macrophage colony stimulating factor; FSA, fibrosarcoma; i.t., intratumoral; p.t., peritumoral; HT, hyperthermia; RX, radiotherapy; SX, surgical excision; d, days.

**Table 3:** Equine cancer immunogene therapy trials.

#	Genes	Tumor	Vector	Mode	Results	Authors/year/reference
	Cytokines	Equine				
1	hIL-12	MEL	Naked plasmid	*In vivo* − i.t./ NAT	(*n* = 7) PR: 59 % reduction of injected tumors burden	Heinzerling et al., 2001 [56]
2	eIL-12	MEL	Naked plasmid	*In vivo* − i.t./ NAT	(*n* = 7) feasibility assessed, pharmacokinetics, and pharmacodynamics of biological activity.	Müller et al., 2011 [58]
3	hIL-12/ hIL-18	MEL	Naked plasmid	*In vivo* − i.t./ NAT	Controlled assay. (*n* = 8) hIL-12, 6 PR; (*n* = 9) hIL-18, 5 PR. Both are very well tolerated.	Müller et al., 2011 [57]
4	hIL-2 + hGM-CSF HSV-*tk *	MEL	Plasmid/cationic lipid for HSV-*tk* + GCV. Irradiated xenogeneic hIL-2 and hGM-CSF producing cells made by plasmid lipofection	*In vivo* (SG) − i.t. *Ex vivo* (CPXC) + (TV) − s.c./+SX	(*n* = 1) case report. Reduction of injected tumors burden. Prevention of postsurgical local relapse. Survival >600 d.	Finocchiaro et al., 2009 [55]

hIL-12, human interleukin-12; eIL-12, equine interleukin-12; hIL-18, human interleukin-18; HSV-*tk*, herpes simplex thymidine kinase; hIL-2, human interleukin-2; hGM-CSF, human granulocyte macrophage colony stimulating factor; MEL, melanoma; CPXC, cytokine producing xenogeneic cells; GCV, ganciclovir; SG, suicide gene; TV, tumor vaccine; i.t., intratumoral; s.c., subcutaneous; NAT, no additional treatment during or following gene therapy; SX, surgical excision; d, days.

## References

[1] Rosenberg S. A., Aebersold P., Cornetta K., Kasid A., Morgan R. A., Moen R., Karson E. M., Lotze M. T., Yang J. C., Topalian S. L., Merino M. J., Culver K., Miller A. D., Blaese R. M., Anderson W. F. (1990). Gene transfer into humans—immunotherapy of patients with advanced melanoma, using tumor-infiltrating lymphocytes modified by retroviral gene transduction. *The New England Journal of Medicine*.

[2] Quintin-Colonna F., Devauchelle P., Fradelizi D., Mourot B., Faure T., Kourilsky P., Roth C., Mehtali M. (1996). Gene therapy of spontaneous canine melanoma and feline fibrosarcoma by intratumoral administration of histoincompatible cells expressing human interleukin-2. *Gene Therapy*.

[3] Nakamura Y., Wakimoto H., Abe J., Kanegae Y., Saito I., Aoyagi M., Hirakawa K., Hamada H. (1994). Adoptive immunotherapy with murine tumor-specific T lymphocytes engineered to secrete interleukin 2. *Cancer Research*.

[4] Vail D. M., MacEwen E. G. (2000). Spontaneously occurring tumors of companion animals as models for human cancer. *Cancer Investigation*.

[5] Hansen K., Khanna C. (2004). Spontaneous and genetically engineered animal models: use in preclinical cancer drug development. *European Journal of Cancer*.

[6] Glikin G. C., Finocchiaro L. M. E., Andrade R. L., Batista C. I. (2012). Gene therapy for canine cancer: clinical results. *Canine Behavior, Classification and Diseases*.

[7] Finocchiaro L. M. E., Glikin G. C., Gustafsson W. B. (2008). Cancer gene therapy in large animals. *New Gene Therapy and Cancer Research*.

[8] Liu L., Wang S., Shan B., Sang M., Liu S., Wang G. (2010). Advances in viral-vector systemic cytokine gene therapy against cancer. *Vaccine*.

[9] Choi I.-K., Yun C.-O. (2013). Recent developments in oncolytic adenovirus-based immunotherapeutic agents for use against metastatic cancers. *Cancer Gene Therapy*.

[10] Wang W., Li W., Ma N., Steinhoff G. (2013). Non-viral gene delivery methods. *Current Pharmaceutical Biotechnology*.

[11] Pavlin D., Cemazar M., Sersa G., Tozon N. (2012). IL-12 based gene therapy in veterinary medicine. *Journal of Translational Medicine*.

[12] Ohlfest J. R., Freese A. B., Largaespada D. A. (2005). Nonviral vectors for cancer gene therapy: prospects for integrating vectors and combination therapies. *Current Gene Therapy*.

[13] Hogge G. S., Burkholder J. K., Culp J., Albertini M. R., Dubielzig R. R., Keller E. T., Yang N.-S., MacEwen E. G. (1998). Development of human granulocyte-macrophage colony-stimulating factor-transfected tumor cell vaccines for the treatment of spontaneous canine cancer. *Human Gene Therapy*.

[14] Dow S. W., Elmslie R. E., Willson A. P., Roche L., Gorman C., Potter T. A. (1998). In vivo tumor transfection with superantigen plus cytokine genes induces tumor regression and prolongs survival in dogs with malignant melanoma. *Journal of Clinical Investigation*.

[15] Bianco S. R., Sun J., Fosmire S. P., Hance K., Padilla M. L., Ritt M. G., Getzy D. M., Duke R. C., Withrow S. J., Lana S., Matthiesen D. T., Dow S. W., Bellgrau D., Cutter G. R., Helfand S. C., Modiano J. F. (2003). Enhancing antimelanoma immune responses through apoptosis. *Cancer Gene Therapy*.

[16] Bergman P. J., McKnight J., Novosad A. (2003). Long-term survival of dogs with advanced malignant melanoma after DNA vaccination with xenogeneic human tyrosinase: a phase I trial. *Clinical Cancer Research*.

[17] Liao J. C. F., Gregor P., Wolchok J. D., Orlandi F., Craft D., Leung C., Houghton A. N., Bergman P. J. (2006). Vaccination with human tyrosinase DNA induces antibody responses in dogs with advanced melanoma. *Cancer Immunity*.

[18] Grosenbaugh D. A., Leard A. T., Bergman P. J., Klein M. K., Meleo K., Susaneck S., Hess P. R., Jankowski M. K., Jones P. D., Leibman N. F., Johnson M. H., Kurzman I. D., Wolchok J. D. (2011). Safety and efficacy of a xenogeneic DNA vaccine encoding for human tyrosinase as adjunctive treatment for oral malignant melanoma in dogs following surgical excision of the primary tumor. *American Journal of Veterinary Research*.

[19] MacEwen E. G., Kurzman I. D., Vail D. M., Dubielzig R. R., Everlith K., Madewell B. R., Rodriguez C. O., Phillips B., Zwahlen C. H., Obradovich J., Rosenthal R. C., Fox L. E., Rosenberg M., Henry C., Fidel J. (1999). Adjuvant therapy for melanoma in dogs: results of randomized clinical trials using surgery, liposome-encapsulated muramyl tripeptide, and granulocyte macrophage colony-stimulating factor. *Clinical Cancer Research*.

[20] Bergman P. J., Camps-Palau M. A., McKnight J. A. (2006). Development of a xenogeneic DNA vaccine program for canine malignant melanoma at the Animal Medical Center. *Vaccine*.

[21] Manley C. A., Leibman N. F., Wolchok J. D. (2011). Xenogeneic murine tyrosinase DNA vaccine for malignant melanoma of the digit of dogs. *Journal of Veterinary Internal Medicine*.

[22] Ottnod J. M., Smedley R. C., Walshaw R., Hauptman J. G., Kiupel M., Obradovich J. E. (2013). A retrospective analysis of the efficacy of Oncept vaccine for the adjunct treatment of canine oral malignant melanoma. *Veterinary and Comparative Oncology*.

[23] Gyorffy S., Rodriguez-Lecompte J. C., Woods J. P., Foley R., Kruth S., Liaw P. C., Gauldie J. (2005). Bone marrow-derived dendritic cell vaccination of dogs with naturally occurring melanoma by using human gp100 antigen. *Journal of Veterinary Internal Medicine*.

[24] Alexander A. N., Huelsmeyer M. K., Mitzey A., Dubielzig R. R., Kurzman I. D., MacEwen E. G., Vail D. M. (2006). Development of an allogeneic whole-cell tumor vaccine expressing xenogeneic gp100 and its implementation in a phase II clinical trial in canine patients with malignant melanoma. *Cancer Immunology, Immunotherapy*.

[25] Von Euler H., Sadeghi A., Carlsson B., Rivera P., Loskog A., Segall T., Korsgren O., Tötterman T. H. (2008). Efficient adenovector CD40 ligand immunotherapy of canine malignant melanoma. *Journal of Immunotherapy*.

[26] Westberg S., Sadeghi A., Svensson E., Segall T., Dimopoulou M., Korsgren O., Hemminki A., Loskog A. S. I., Tötterman T. H., Von Euler H. (2013). Treatment efficacy and immune stimulation by AdCD40L gene therapy of spontaneous canine malignant melanoma. *Journal of Immunotherapy*.

[27] Riccardo F., Iussich S., Maniscalco L., Lorda-Mayayo S., La Rosa G., Arigoni M., De Maria R., Gattino F., Lanzardo S., Lardone E., Martano M., Morello E., Prestigio S., Fiore A., Quaglino E., Zabarino S., Ferrone S., Buracco P., Cavallo F. (2014). CSPG4-specific immunity and survival prolongation in dogs with oral malignant melanoma immunized with human CSPG4 DNA. *Clinical Cancer Research*.

[28] Finocchiaro L. M. E., Fiszman G. L., Karara A. L., Glikin G. C. (2008). Suicide gene and cytokines combined nonviral gene therapy for spontaneous canine melanoma. *Cancer Gene Therapy*.

[29] Finocchiaro L. M. E., Glikin G. C. (2008). Cytokine-enhanced vaccine and suicide gene therapy as surgery adjuvant treatments for spontaneous canine melanoma. *Gene Therapy*.

[30] Finocchiaro L. M. E., Glikin G. C. (2012). Cytokine-enhanced vaccine and suicide gene therapy as surgery adjuvant treatments for spontaneous canine melanoma: 9 years of follow-up. *Cancer Gene Therapy*.

[31] Finocchiaro L. M. E., Fondello C., Gil-Cardeza M. L. Cytokine-enhanced vaccine and interferon-*β* plus suicide gene therapy as surgery adjuvant treatments for spontaneous canine melanoma.

[32] Ehrhart N. (2005). Soft-tissue sarcomas in dogs: a review. *Journal of the American Animal Hospital Association*.

[33] Thamm D. H., Kurzman I. D., Macewen E. G., Feinmehl R., Towell T. L., Longhofer S. L., Johnson C. M., Geoly F. J., Stinchcomb D. T. (2003). Intralesional lipid-complexed cytokine/superantigen immunogene therapy for spontaneous canine tumors. *Cancer Immunology, Immunotherapy*.

[34] Finocchiaro L. M. E., Villaverde M. S., Gil-Cardeza M. L., Riveros M. D., Glikin G. C. (2011). Cytokine-enhanced vaccine and interferon-β plus suicide gene as combined therapy for spontaneous canine sarcomas. *Research in Veterinary Science*.

[35] Mayer M. N., Grier C. K. (2006). Palliative radiation therapy for canine osteosarcoma. *Canadian Veterinary Journal*.

[36] Dow S., Elmslie R., Kurzman I., MacEwen G., Pericle F., Liggitt D. (2005). Phase I study of liposome-DNA complexes encoding the interleukin-2 gene in dogs with osteosarcoma lung metastases. *Human Gene Therapy*.

[37] Finocchiaro L. M. E., Spector A., Rossi U. A., Butler E. J. (2012). The potential of suicide plus immune gene therapy for treating osteosarcoma: the experience on canine veterinary patients. *Sarcoma: Symptoms, Causes and Treatments*.

[38] Modiano J. F., Bellgrau D., Cutter G. R., Lana S. E., Ehrhart N. P., Ehrhart E., Wilke V. L., Charles J. B., Munson S., Scott M. C., Pozniak J., Carlson C. S., Schaack J., Duke R. C. (2012). Inflammation, apoptosis, and necrosis induced by neoadjuvant fas ligand gene therapy improves survival of dogs with spontaneous bone cancer. *Molecular Therapy*.

[39] Marconato L., Gelain M. E., Comazzi S. (2013). The dog as a possible animal model for human non-Hodgkin lymphoma: a review. *Hematological Oncology*.

[40] Turek M. M., Thamm D. H., Mitzey A., Kurzman I. D., Huelsmeyer M. K., Dubielzig R. R., Vail D. M. (2007). Human granulocyte-macrophage colony-stimulating factor DNA cationic-lipid complexed autologous tumour cell vaccination in the treatment of canine B-cell multicentric lymphoma. *Veterinary and Comparative Oncology*.

[41] Lawman M., Eidizadeh S., Selmon C., Kane C., Xigacos L., Kaufman L., Shaw N., Lawman P. (2008). Anti-tumor response induced by autologous cancer vaccine in canine lymphoma. *Cancer Therapy*.

[42] Peruzzi D., Gavazza A., Mesiti G., Lubas G., Scarselli E., Conforti A., Bendtsen C., Ciliberto G., La Monica N., Aurisicchio L. (2010). A vaccine targeting telomerase enhances survival of dogs affected by B-cell lymphoma. *Molecular Therapy*.

[43] Gavazza A., Lubas G., Fridman A., Peruzzi D., Impellizeri J. A., Luberto L., Marra E., Roscilli G., Ciliberto G., Aurisicchio L. (2013). Safety and efficacy of a genetic vaccine targeting telomerase plus chemotherapy for the therapy of canine B-cell lymphoma. *Human Gene Therapy*.

[44] Sorenmo K. U., Krick E., Coughlin C. M., Overley B., Gregor T. P., Vonderheide R. H., Mason N. J. (2011). CD40-activated B cell cancer vaccine improves second clinical remission and survival in privately owned dogs with non-Hodgkin's lymphoma. *PLoS ONE*.

[45] Reed S. D., Fulmer A., Buckholz J., Zhang B., Cutrera J., Shiomitsu K., Li S. (2010). Bleomycin/interleukin-12 electrochemogene therapy for treating naturally occurring spontaneous neoplasms in dogs. *Cancer Gene Therapy*.

[46] Pluhar G. E., Grogan P. T., Seiler C., Goulart M., SantaCruz K. S., Carlson C., Chen W., Olin M. R., Lowenstein P. R., Castro M. G., Haines S. J., Ohlfest J. R. (2010). Anti-tumor immune response correlates with neurological symptoms in a dog with spontaneous astrocytoma treated by gene and vaccine therapy. *Vaccine*.

[47] Cemazar M., Sersa G., Pavlin D., Tozon N., You Y. (2011). Intramuscular IL-12 electrogene therapy for treatment of spontaneous canine tumors. *Targets in Gene Therapy*.

[48] Pavlin D., Cemazar M., Cör A., Sersa G., Pogacnik A., Tozon N. (2011). Electrogene therapy with interleukin-12 in canine mast cell tumors. *Radiology and Oncology*.

[49] Nande R., Di Benedetto A., Aimola P., de Carlo F., Carper M., Claudio C. D., Denvir J., Valluri J., Duncan G. C., Claudio P. P. (2012). Targeting a newly established spontaneous feline fibrosarcoma cell line by gene transfer. *PLoS ONE*.

[50] Jourdier T. M., Moste C., Bonnet M. C., Delisle F., Tafani J. P., Devauchelle P., Tartaglia J., Moingeon P. (2003). Local immunotherapy of spontaneous feline fibrosarcomas using recombinant poxviruses expressing interleukin 2 (IL2). *Gene Therapy*.

[51] Siddiqui F., Li C.-Y., LaRue S. M., Poulson J. M., Avery P. R., Pruitt A. F., Zhang X., Ullrich R. L., Thrall D. E., Dewhirst M. W., Hauck M. L. (2007). A phase I trial of hyperthermia-induced interleukin-12 gene therapy in spontaneously arising feline soft tissue sarcomas. *Molecular Cancer Therapeutics*.

[52] Jahnke A., Hirschberger J., Fischer C., Brill T., Köstlin R., Plank C., Küchenhoff H., Krieger S., Kamenica K., Schillinger U. (2007). Intra-tumoral gene delivery of feIL-2, feIFN-*γ* and feGM-CSF using magnetofection as a neoadjuvant treatment option for feline fibrosarcomas: a phase-I study. *Journal of Veterinary Medicine Series A: Physiology Pathology Clinical Medicine*.

[53] Hüttinger C., Hirschberger J., Jahnke A., Köstlin R., Brill T., Plank C., Küchenhoff H., Krieger S., Schillinger U. (2008). Neoadjuvant gene delivery of feline granulocyte-macrophage colony-stimulating factor using magnetofection for the treatment of feline fibrosarcomas: a phase I trial. *Journal of Gene Medicine*.

[54] Phillips J. C., Lembcke L. M. (2013). Equine melanocytic tumors. *Veterinary Clinics of North America—Equine Practice*.

[55] Finocchiaro L. M. E., Riveros M. D., Glikin G. C. (2009). Cytokine-enhanced vaccine and suicide gene therapy as adjuvant treatments of metastatic melanoma in a horse. *Veterinary Record*.

[56] Heinzerling L. M., Feige K., Rieder S., Akens M. K., Dummer R., Stranzinger G., Moelling K. (2001). Tumor regression induced by intratumoral injection of DNA coding for human interleukin 12 into melanoma metastases in gray horses. *Journal of Molecular Medicine*.

[57] Müller J., Feige K., Wunderlin P., Hödl A., Meli M. L., Seltenhammer M., Grest P., Nicolson L., Schelling C., Heinzerling L. M. (2011). Double-blind placebo-controlled study with interleukin-18 and interleukin-12-encoding plasmid DNA shows antitumor effect in metastatic melanoma in gray horses. *Journal of Immunotherapy*.

[58] Müller J.-M. V., Wissemann J., Meli M. L., Dasen G., Lutz H., Heinzerling L., Feige K. (2011). In vivo induction of interferon gamma expression in grey horses with metastatic melanoma resulting from direct injection of plasmid DNA coding for equine interleukin 12. *Schweizer Archiv fur Tierheilkunde*.

[59] Laborda E., Puig-Saus C., Rodriguez-García A., Moreno R., Cascalló M., Pastor J., Alemany R. (2014). A pRb-responsive, RGD-modified, and hyaluronidase-armed canine oncolytic adenovirus for application in veterinary oncology. *Molecular Therapy*.

[60] Kamstock D., Guth A., Elmslie R. (2006). Liposome-DNA complexes infused intravenously inhibit tumor angiogenesis and elicit antitumor activity in dogs with soft tissue sarcoma. *Cancer Gene Therapy*.

[61] U'Ren L. W., Biller B. J., Elmslie R. E., Thamm D. H., Dow S. W. (2007). Evaluation of a novel tumor vaccine in dogs with hemangiosarcoma. *Journal of Veterinary Internal Medicine*.

[62] Paoloni M. C., Khanna C. (2007). Comparative oncology today. *Veterinary Clinics of North America: Small Animal Practice*.

[63] Rowell J. L., McCarthy D. O., Alvarez C. E. (2011). Dog models of naturally occurring cancer. *Trends in Molecular Medicine*.

[64] Ranieri G., Gadaleta C. D., Patruno R. (2013). A model of study for human cancer: spontaneous occurring tumors in dogs. Biological features and translation for new anticancer therapies. *Critical Reviews in Oncology/Hematology*.

[65] Porrello A., Cardelli P., Spugnini E. P. (2004). Pet models in cancer research: general principles. *Journal of Experimental and Clinical Cancer Research*.

[66] Khanna C., London C., Vail D., Mazcko C., Hirschfeld S. (2009). Guiding the optimal translation of new cancer treatments from canine to human cancer patients. *Clinical Cancer Research*.

